# Realization of Microfluidic Preconcentrator for N-Pentane Traces Impurities from the Gaseous Media

**DOI:** 10.3390/ma15228090

**Published:** 2022-11-15

**Authors:** Vladimir Platonov, Prachi Sharma, Mikhail Ledyaev, Maria A. Anikina, Nikolay Alekseevich Djuzhev, Maksim Yuryevich Chinenkov, Nishant Tripathi, Sania Parveen, Rafiq Ahmad, Vladimir Pavelyev, Ammar A. Melaibari

**Affiliations:** 1 Samara National Research University, 34, Moskovskoye Shosse, 443086 Samara, Russia; 2 School of Electronics Engineering (SENSE), Vellore Institute of Technology (VIT), Vellore 632014, India; 3 Scientific Research Institute of the Department of Gas Processing, Hydrogen and Special Technologies, Samara State Technical University, 443100 Samara, Russia; 4 National Research University of Electronic Technology (MIET), 124498 Moscow, Russia; 5 Central Instrumentation Facility, Jamia Millia Islamia, New Delhi 110025, India; 6 Centre for Nanoscience and Nanotechnology, Jamia Millia Islamia, New Delhi 110025, India; 7 Center of Nanotechnology, King Abdulaziz University, Jeddha 22254, Saudi Arabia

**Keywords:** microfabricated systems, MEMS, microfluidic preconcentrators

## Abstract

In this paper, we present the work of designing and fabricating a new generation of microelectromechanical systems (MEMS) based microfluidic preconcentrators (MFP) for volatile organic compounds (VOCs) quantification. The main objective of this work is to quantify the n-pentane impurities using MFP for sample preparation. The MFP was analyzed using Hewlett-Packard 5890 gas chromatography, having a flame ionization detector under isothermal conditions. The proposed MFP system includes two-microfluidic preconcentrators for continuous action and a system of four 3/2 solenoid valves with a control unit. Microfluidic preconcentrators were placed on metal plates and have circular channels filled with Al_2_O_3_ (50 μm), n-octane ResSil-C (80/100 mesh) sorbents of one nature and are hyphenated with the Peltier elements to regulate the temperature of sorption and desorption. The n-pentane quantitative determination was carried out using a calibration plot of gas mixtures on a successive dilution with the nitrogen. This study shows that the microfluidic preconcentrator system with Al_2_O_3_ and n-Octane ResSil-C sorbent concentrates the n-pentane traces up to 41 to 47 times from the gas mixture with the standard deviation of ≤5%. It has been observed that the n-octane ResSil-C based MFC shows very fast response (<5 min) and stability up to 300 cycles.

## 1. Introduction

Volatile organic compounds (VOCs) are the organic compounds with high vapor pressure and low affinity for water. These compounds contain a large group of chemicals/substances—for example, unsaturated hydrocarbons, alkanes, alcohols, benzenes, esters, aldehydes ketones, halogenated hydrocarbons, etc. [1,2]. When exposed to the humans, most of the VOCs show toxicological properties and mutagenicity, carcinogenicity, and teratogenicity [3,4,5,6,7]. Among all VOCs, n-pentane, which evolves from the reaction involving omega-6 fatty acids, is a promising predictor of lipid peroxidation [4]. The concentrations of many exhaled hydrocarbons are very low, i.e., in the pico-molar range. Therefore, the analysis of such types of VOCs is quite tedious and requires pre-concentration steps for the quantification in various samples [8,9,10,11].

To this date, several sample preparation methods have been employed to isolate and concentrate VOCs, such as solid phase extraction (SPE), liquid extraction, simultaneous steam distillation, and head-space (HS) analysis. Among these, HS analysis is used either alone or combined with other method, such as SPE, which is the mostly used procedure due to its minimal sample treatment requirement and, thus, reduces the formation of artifactual volatile compounds. Head space and dynamic headspace gas chromatography are utilized to determine the volatile compounds in infant formulas [12].

A solid phase extraction consists of bringing a liquid or gaseous test sample in contact with a solid phase, whereby the analyte is selectively adsorbed on the surface of the solid phase. The basic principles of SPE involve the distribution of dissolved species between two phases. SPE involves the dispersion of the analyte between a liquid (sample medium) and a solid (adsorbent) phase. This technique allows the enrichment and purification of the analytes on a solid adsorbent through adsorption from the solution. This method is good for high throughput performance and also does not need extended sample preparations. Hence, it saves time. Additionally, it is simple, effective, reproducible, inexpensive, it shows minimal interference (are selective during analysis) from sample matrix, and it does not use solvents for extraction [13,14]. All mentioned techniques for the development of preconcentrators show excellent metrological properties but also have certain limits. However, the requirement of replacement for sorption cartridges is a big challenge for existing technology. Apart from this, after every operation, devices need to be cooled down. All these shortcomings increase the operational cost, as well as time.

The emerging trend of modern analytical instrumentation is the creation of mobile gas chromatographic (GC) systems that allow continuous monitoring of natural and man-made objects. Additionally, these systems can provide non-invasive diagnostics in the early stages of the disease by exhaled air with minimal time and cost, including the stages of sampling and sample preparation. To eliminate some of the drawbacks of conventional laboratory GC facility, scientists directed significant efforts towards the development of micro-total analysis systems. Since the mid-1980s, these systems, which are built from silicon microstructural components, have been progressively developing. Their potential was in miniaturization, lower power consumption, and greater analytical capabilities. Further development of this technology lies in the creation of microelectromechanical systems (MEMS). These are miniature mechanical devices, namely, gas micro chromatographs equipped with a micromechanical concentrator, a GC column with micromechanical processing, and a microdetector [4,12,13,14,15,16].

Nowadays, microfluidic systems (MFS) are used as microconcentrators in gas analysis, which became widespread owing to their small size and the possibility of using them in portable installations, as well as their high sensitivity, reaching up to 0.5 ppbv (parts per billion by volume), and their relatively short time for one analysis cycle (up to 0.2 min) [17]. In MFPs, the rate of flow is directly proportional to the difference of pressure applied to the two ends of the channel, and it also depends on the geometry of the channel used and the viscosity of the fluid in consideration. Active components, such as valves, are also commonly found in microfluidic systems, allowing modulation of the fluid flow. In GC, microfluidic systems on the planes of glass, metal, and silicon plates, as well as polymer materials, are used in the separation of chromatographic columns and devices for concentration. The inner surface of microchannels is modified with various adsorbents and polymer liquids, which vary depending on the sorbates separated on the column [18]. Thermal desorption tubes containing a sorbent, automated devices for solid phase microextraction (SPM), and microconcentrators based on injection needles are widely used as devices for the concentration of analytes based on microfluidics.

To date, in the practice of mass diagnostics of exhaled air, overall gas chromatography equipment is used, the use of which is possible only in laboratory conditions. The development of non-invasive diagnostics is also constrained by the lack of a device that performs sample preparation in automatic mode, which excludes contamination and sample loss. The results of this work open up the possibility of creating a portable device that allows for rapid and continuous analysis of exhaled air samples.

It is important to emphasize that small-sized devices made on an integral basis have found their application, among other things, to the preparation of calibration gas mixtures containing both organic and inorganic components. Such dynamic methods for the standard gas media preparation are considered in detail [12,19,20,21,22]. Thus, the implementation of microfluidics and MEMS make it possible to create a new type of not only GC columns, but also a new generation of microconcentrators on plates made of various materials and devices for producing gas mixtures for calibration. In the present work, to further improve the MFP system, we design the unique type preconcentrator for n-pentane by using Al_2_O_3_ and n-octane ResSil-C sorbents. The advanced Peltier elements have been utilized to regulate the temperature of sorption and desorption. The device was optimized and tested for both sorbents for its stability and efficiency. The results have been compared with the reported data.

## 2. Experimental

### 2.1. Materials

A calibrated solution of hydrocarbons has been prepared by using the gas mixture generator GGS-03-03 (Monitoring LLC, Saint Petersburg, Russia), n-pentane, Al_2_O_3_, and n-octane ResSil-C, which were bought from Sigma-Aldrich (St. Louis, MO, USA).

### 2.2. Device Design for the Microextraction, Fabrication, and Operation Principle

**Figure 1:**
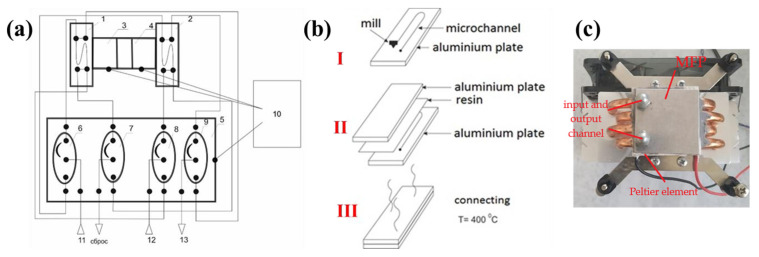
(**a**) Shows the schematic diagram for as-prepared MFP. The notations are as follows: 1, 2—microfluidic preconcentrators; 3, 4—Peltier elements; 5—flat aluminum plate; 6–9—3/2 electrovalves; 10—control unit; 11, 12—gas inlet and outlet; 13—chromatographic column. (**b**) Shows the fabrication steps followed in preparation of MFP, and (**c**) shows the optical image of final device.

To carry out the preconcentration of n-pentane from air, MFP was developed and manufactured at Samara University [23]. The proposed modality has remarkable features, such as smaller dimensions and low power consumption. It greatly simplifies the procedure for analyzing VOCs in various gaseous media due to the continuous process of analyzing concentrated impurities. Figure 1 shows the schematic diagram of as-prepared MFP, fabrication steps, and the optical image for the final product. The MFP device is comprised of two microfluidic preconcentrators located on Peltier elements for temperature adjustment during the concentration and desorption stages. These preconcentrators are filled with an adsorbent that has the ability to remove impurities from VOCs even at very low level of gaseous media. The channels for the sorption tubes have identical geometry and are made up of metal plates. A system of four 3/2 electrovalves (EV) were used to implement a continuous analysis process. The schematic diagram for the MFP is shown Figure 1a. The MEMS concentrator is a serpentine microchannel of circular cross-section 360 mm long and 0.4 mm in diameter, which was placed upon a metal aluminum plate. The choice of the material used is due to its properties, namely, inertness to the selected analytes and high thermal conductivity, which make it possible to thermostat the system. The manufacture of MEMS concentrators consisted of the formation of channels of the selected configuration on pre-polished aluminum plates by micromilling. Then, the microchannels were mechanically filled with the selected sorbents. The filling process was controlled using a optical microscope (Carl Zeiss Micro Imagine Gmb H 37081, Oberkohen, Germany). After that, the plane of the MEMS concentrators was covered with a polished aluminum plate and sealed by thermal bonding through a polymethyl methacrylate film at a temperature of 130 °C under vacuum (Figure 1b).

In as-prepared MFP, microfluidic preconcentrators 1 and 2 were made in the form of a microfluidic preconcentrator column using the technology of metal powders laser sintering. These columns are circular cross-section channels with a length of 360 mm and a diameter of 0.4 mm. In these columns, Al_2_O_3_ (50 μm) and n-octane ResSil-C (80/100 mesh) were used as adsorbents. Peltier elements 3 and 4 were used to heat and cool the thermal desorber. 3/2 electrovalves (EV)-6–9 and control unit-10 were placed on a flat aluminum plate-5. The analyzed gas enters the system through inlets 11 and 12 and comes out through outlet 13 to the chromatographic column (Figure 1a). The MFP operates through the principle that the gas is allowed to pass through inlet 11 to reach electrovalve 6 and alternatively provides input to microfluidic preconcentrators 1 or 2, resulting in a switch of electrovalve 7 for concentrators 1 or 2, which leads to the output to the dump line. Electrovalve 8 connects the outlet of concentrarors 1 or 2 to the chromatographic column. Electrovalve 9 connects the inlet concentrators 1 or 2 to the chromatographic column. Control unit 10, according to a given program, switches electrovalve (EV) 6 EV 9 and includes Peltier elements for temperature control (Figure 1b).

Quantitative assessment of the content of n-pentane at the outlet of the MPF was carried out by the method of absolute calibration using standard gas mixtures with concentrations of 0.1, 0.5, 1.0, and 10 mg/m^3^, a Hewlett-Packard 5890 gas chromatograph (Conquer Scientific, Poway, CA, USA) with a flame ionization detector, and a DB-5 capillary column (length 30 m) (inner diameter 0.32 mm and film thickness 0.5 µm).

### 2.3. Conditions for Concentration and GC Analysis

The sorption of n-pentane was carried out on sorbents Al_2_O_3_, as well as on n-octane ResSil-C8, at a temperature of +5 °C. The flow rate of n-pentane through the device has been used as a thermal desorber (TD) (5 mL/min). Desorption temperature was +75 °C. Volume of the passed gas mixture was 20 mL.

To evaluate the operation of MEMS/TD, a Hewlett-Packard 5890 gas chromatograph, having a flame ionization detector and a DB-5 capillary column (30 m long) and a 0.32 mm inner diameter with a film thickness of 0.5 μm, were used. The content of n-pentane in the HS, before and after concentration, was determined in an isothermal mode; the column was maintained at a temperature (Tc) of 50 °C. Nitrogen was used as the carrier gas; the flow rate at the column outlet was Fc = 1 cm^3^/min, and the rate of discharge observed was 1/50.

## 3. Results and Discussion

n-pentane was chosen as the object of the study because it is a promising predictor of lipid metabolism dysfunctions determined in exhaled alveolar air. A calibration gas mixture, “n-pentane in nitrogen”, with a concentration of 10 mg/m^3^, was used as the initial mixture. Model gas mixtures of “n-pentane-nitrogen”, with concentrations of 0.1, 0.5, 1.0, and 10 mg/m^3^, were prepared using a gas mixture generator GGS-03-03. n-pentane concentration from model gas mixtures with a known analyte content using MFP was carried out according to the scheme shown in Figure 1a. n-pentane sorption was carried out on sorbents Al_2_O_3_ and n-octane ResSil-C at a temperature of +5 °C. To do this, the n-pentane-nitrogen gas mixture was passed through MFP at a constant rate of 5 mL/min. The desorption temperature was +75 °C. The passed gas mixture volume was 20 mL. The target component quantitative determination was carried to evaluate the concentration efficiency. The concentration coefficient was determined using the following mathematical equation:

(1) $$\mathrm{Kc}=\frac{C_{des}}{C_{0}}$$

where *C*_0_ is the concentration of the analyte in the initial mixture, mg/m^3^, and *C_des_* is the concentration of the analyte in the mixture obtained after desorption, mg/m^3^. *C_des_* was determined using a calibration dependence constructed from the calibration mixtures series chromatography results of n-pentane in nitrogen. The calibration function is linear. The expanded uncertainty in measuring the n-pentane concentration, *U_Cdes_*, was calculated for k = 2 at P (confidence interval) = 0.95 (see Table 1).

The calculation procedure and designation were performed in accordance with previously published reports [24,25]. Table 2 shows the results of the n-pentane concentration using MFP filled with various sorbents. From the calculated data (Table 2), it is clearly reflected that the MFP systems filled with Al_2_O_3_ and n-octane ResSil-C are capable to concentrate the n-pentane at a factor of 41–47 times. Thus, the investigated MFP can be used to concentrate components from gas mixtures with a concentration of up to 10 mg/m^3^.

**Figure 2:**
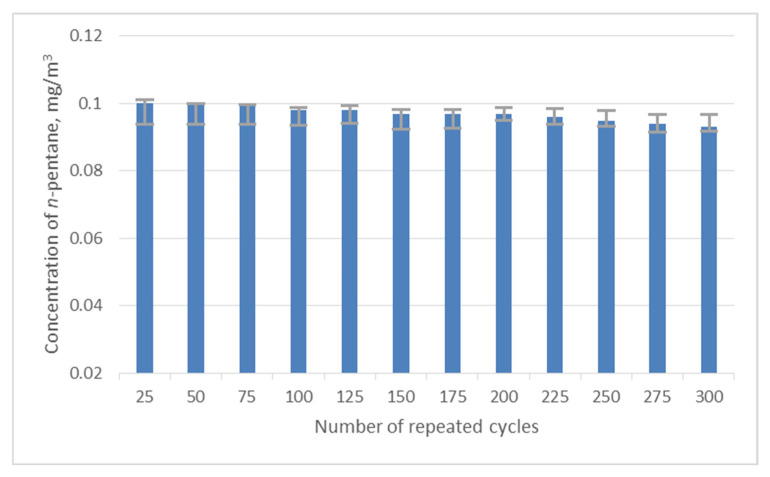
Graph showing the concentration of the n-pentane as the function of number of cycles for the n-octane ResSil-C sorbent.

**Figure 3:**
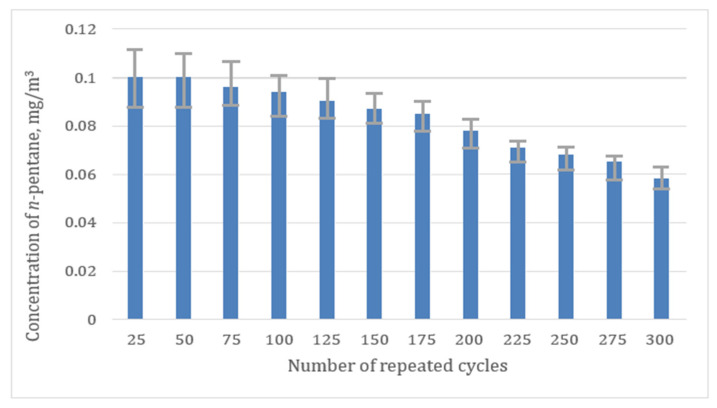
Graph showing the dependence of the n-pentane concentration on the number of cycles for the Al_2_O_3_ sorbent.

When carrying out a continuous sorption–desorption procedure, an important characteristic is the duration of the adsorbent operation under the selected analysis conditions (temperature and flow rate of the carrier gas). To make it clearer, the duration for the sorption–desorption process has been observed for repeating cycles with both types of adsorbents (Al_2_O_3_ and n-octane ResSil-C), and the data have been recorded for 300 cycles. Figure 2 and Figure 3 represent the observation for the average concentration (0.1 mg/m^3^) of n-pentane obtained from ten values depending on a function of repeating cycles. It is clear from the observations that the chemically modified (with non-polar groups) ResSil-C shows the best stability over the 300 cycles (Figure 2). No change in the concentration of n-pentane has been detected over the 300 cycles. In the case of Al_2_O_3_, the concentration of n-pentane has decreased slowly (Figure 3); the maximum standard deviation has been observed as 5% over 300 measurement cycles. A significant decrease in the concentration of Al_2_O_3_ is probably associated with its emerging chemical modifications due to the adsorption of water on its surface. The investigated MFP based on n-octane ResSil-C was used to calibrate a gas chromatograph under identical conditions to sample preparations. Previously, it was shown that this technique significantly reduces the determination error [12,26].

To assess the proposed approach quality, the “introduced-found” method was used. The obtained results and the estimated standard deviation are presented in Table 2.

**Figure 4:**
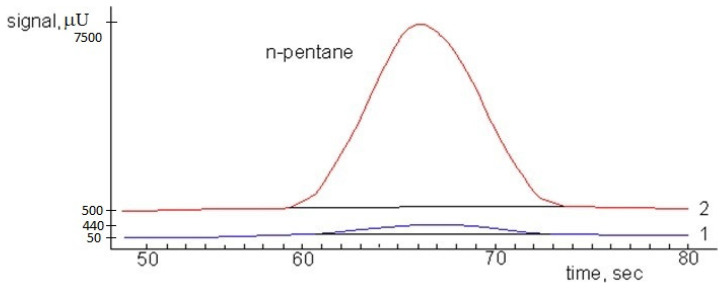
Chromatogram analysis of a mixture of air and n-pentane (concentration 0.5 mg/m^3^): 1—analysis of the original gas mixture; 2—analysis of the mixture after concentration.

Demonstrated data show that the standard deviation from the true value does not exceed the methodological error. It indicates the correctness of the approach used. Some experimentally determined concentration underestimation is associated with the presence of unaccounted uncertainty sources in the concentration stage. After optimization of as-prepared MFP for best results, the device was used for determining the n-pentane in open air (Figure 4).

Figure 4 shows the chromatograms for the determination of n-pentane in air. The chromatogram of the initial mixture with a concentration of 0.5 mg/m^3^ is highlighted in blue, and the chromatogram for the determination of pentane in air with a concentration of 0.5 mg/m^3^ using MFP is highlighted in red. It can be seen that the use of MFP can significantly increase the ensitivity of the instrument in relation to the analyte. The results of this work were compared with the reported data (see Table 3); it is clearly observable from the literature survey that as-prepared MFP gives better results with less analysis time.

Based on the literature survey (see Table 3), the use of the as-developed device provides the lowest LOD values as compared to known methods [28,29] and, at the same time, allows the detection of rather low concentrations of the analyte. Additionally, by using the as-developed device, it becomes possible to reduce the sample preparation time to 5 min, which meets modern requirements for rapid analysis. Thus, MFC based on n-octane ResSil-C has the best characteristics and can be used to prepare exhaled air for analysis.

## 4. Conclusions

In the present work, a MEMS-based highly efficient microfluidic preconcentrator has been realized for n-pentane substances. The device has been optimized for two adsorbents (i.e., Al_2_O_3_ and n-octane ResSil-C). It has been concluded that n-octane ResSil-C is a better and more effective adsorbent as compared to Al_2_O_3_. n-octane ResSil-C-based MFC is capable of enhancing the concentration of n-pentane up to 47 times with a maximum standard deviation of about 5%. It has been observed that the n-octane ResSil-C-based MFC shows very fast response (<5 min) and stability up to 300 cycles. The performance of devices was also successfully recorded to detect n-pentane from climate. An as-prepared MFC system can be used to concentrate the n-pentane biomarker with a concentration of up to 10 mg/m^3^ from the air exhaled by a human in order to improve the accuracy of non-invasive diagnostics of metabolic disorders.

## Figures and Tables

**Table 1:** Obtained results for n-pentane concentration by using as-developed MFP.

Sorbent Used in Microfluidic Preconcentrator	n-Pentane Concentration in the Model Gas Mixture, *C_true_* ± *U_Ctrue_*, mg/m^3^	Experimentally Determined n-Pentane Concentration, *C_MV_* ± *U*, mg/m^3^	Deviation from True * Value, mg/m^3^
n-Octane ResSil-C	0.100 ± 0.004	0.09 ± 0.02	0.01
	1.00 ± 0.04	0.92 ± 0.20	0.08
	10.0 ± 0.4	9.8 ± 0.6	0.2

*—the true n-pentane concentration value in the model mixture by gas chromatography under isothermal mode on a Hewlett-Packard 5890 gas chromatograph with a flame ionization detector. The analysis mode is similar to the mode when evaluating the proposed MFP.

**Table 2:** Comparative evaluation between two methods used for analysis of n-pentane in air.

Sorbent	Initial n-Pentane Concentration *C*_0_ ± *U_C_*_0_, mg/m^3^	Final n-Pentane Concentration *C_des_* ± *U_Cdes_*, mg/m^3^	Concentrat ion Factor, K_c_
Al_2_O_3_	0.100 ± 0.004	4.20 ± 0.4	42.0
	1.00 ± 0.04	42.1 ± 0.3	42.1
	10.0 ± 0.4	416.6 ± 15.1	41.7
n-octane ResSil-C	0.100 ± 0.004	4.90 ± 0.5	49.0
	1.00 ± 0.04	45.8 ± 0.3	45.8
	10.0 ± 0.4	464.2 ± 18.2	46.4

**Table 3:** Table shows the comparative data with reported ones.

S. No.		Pre-Concentrator		Target Compounds	Analysis Time/Cycle (min)	LOD (ppb)	Detector	Ref.
	Materials	Adsorbent	Heating system					
1		ResSil-B		VOC	35	0.1–1.6 (BTEX)	PID	[27]
2	Al	Basolite C300	Cartridge	BTEX	19	0.002–0.011 (BTEX)	PID	[28]
3	Si-glass	Tenax	Cr/N	VOC	<12	~25 (TEX)	TCD	[29]
4	Al	n-octane ResSil-C	Peltier elements	n-pentane	<5	0.1 (n-pentane)	PID	Our work

## Data Availability

No data need to be presented. This is a normal research article, which was carried out in the University. All data generated or analyzed during this study are included in this article.

## References

[1] Cikach F.S., Dweik R.A. (2012). Cardiovascular biomarkers in exhaled breath. Prog. Cardiovasc. Dis..

[2] Hunter G.W., Akbar S., Bhansali S., Daniele M., Erb P.D., Johnson K., Liu C.C., Miller D., Oralkan O., Hesketh P.J. (2020). Editors’ Choice—Critical Review—A critical review of solid-state gas sensors. J. Electrochem. Soc..

[3] Siegal M.P., Mowry C.D., Pfeifer K.B., Gallis D.F.S. (2015). Detecting trihalomethanes using nanoporous-carbon coated surface-acoustic-wave sensors. J. Electrochem. Soc..

[4] Lemoyne M., van Gossum A., Kurian R., Ostro M., Axler J., Jeejeebhoy K.N. (1987). Breath pentane analysis as an index of lipid peroxidation: A functional test of vitamin E status. Am. J. Clin. Nutr..

[5] Sagnik D., Mrinal P. (2020). Review—Non-invasive monitoring of human health by exhaled breath analysis: A comprehensive review. J. Electrochem. Soc..

[6] Horrillo M.C., Getino J., Arés L., Robia J.I., Gutiérrez I.S.F.J. (1998). Measurements of VOCs with a semiconductor electronic nose. J. Electrochem. Soc..

[7] Masikini M., Chowdhury M., Nemraoui O. (2020). Review—Metal oxides: Application in exhaled breath acetone chemiresistive sensors. J. Electrochem. Soc..

[8] Akita T., Otsuka Y., Hayase M. (2020). Microfluidic device for in situ observation of bottom-up copper electrodeposition in a TSV-Like structure. J. Electrochem. Soc..

[9] Rodsud S., Limbut W. (2019). A simple electrochemical sensor based on graphene nanoplatelets modified glassy carbon electrode (GrNPs/GCE) for highly sensitive detection of yohimbine (YOH). J. Electrochem. Soc..

[10] Hatami E., Ashraf N., Zavar M.H.A. (2019). On-chip electrochemical sensing of propylparaben as an endocrine disruptor model using a disposable gold platform modified with phosphomolybdate doped polypyrrole and nanodiamond. J. Electrochem. Soc..

[11] Zhang F., Co A.C. (2020). Rapid product analysis for the electroreduction of CO_2_ on heterogeneous and homogeneous catalysts using a rotating ring detector. J. Electrochem. Soc..

[12] Platonov I.A., Kolesnichenko I.N., Lange P.K. (2017). Chromatographic-desorption method for preparing calibration gas mixtures of volatile organic compounds. Meas. Tech..

[13] Arthur C.L., Pawliszyn J. (1990). Solid phase microextraction with thermal desorption using fused silica optical fibers. Anal. Chem..

[14] Zhang Z., Yang M.J., Pawliszyn J. (1994). Solid-phase microextraction. a solvent-free alternative for sample preparation. Anal. Chem..

[15] Sidelnikov N.V., Nikolaeva O.A., Platonov I.A., Parmon V.N. (2016). Gas chromatography of the future: Columns whose time has come. Adv. Chem..

[16] Kumar S., Pavelyev V., Tripathi N., Platonov V., Sharma P., Ahmad R., Mishra P., Khosla A. (2020). Recent advances in the development of carbon nanotubes based flexible sensors. J. Electrochem. Soc..

[17] Lourenco C., Turner C. (2014). Breath analysis in disease diagnosis: Methodological considerations and applications. Metabolites.

[18] Platonov I.A., Platonov V.I., Arutyunov Y.I. (2016). Planar microchromatographic columns for gas chromatography. J. Anal. Chem..

[19] Platonov I., Rodinkov O.V., Gorbacheva A.R., Moskvin L.N., Kolesnichenko I.N. (2018). Methods and devices for the preparation of standard gas mixtures. J. Anal. Chem..

[20] Platonov I., Kolesnichenko I.N., Lange P.K. (2018). Using chromatography–desorption method of manufacturing gas mixtures for analytical instruments calibration. J. Phys. Conf. Ser..

[21] Slominska M., Konieczka P., Namiesnik J. (2010). Standard gas mixtures—Indispensable reference materials in the analysis of gaseous media. Trends Anal. Chem..

[22] Fijalo C., Dymerski T., Gebicki J., Namiesnik J. (2016). Devices for the production of reference gas mixtures. Crit. Rev. Anal. Chem..

[23] Platonov I.A., Platonov V.I., Ledyaev M.E., Voron S.V. (2021). The use of a microthermal desorber for the concentration of trace amounts of hydrocarbons in the air. Sorbtsionnye Khromatograficheskie Protsessy.

[24] Ellison S.L.R., Williams A. (2000). Guide Quantifying Uncertainty in Analytical Measurement.

[25] (2008). Uncertainty of Measurement—Part 3: Guide to the Expression of Uncertainty in Measurement. Declared 2008-10-01.

[26] Kayutkina N.I., Platonov I.A., Bulanova A.V. (2004). Sample pretreatment and chromatographic determination of benz[a]pyrene in wastewater. Russ. J. Appl. Chem..

[27] Skog K.M., Xiong F., Kawashima H., Doyle E., Soto R., Gentner D.R. (2019). Compact, automated, inexpensive, and field-deployable vacuum-outlet gas chromatograph for trace-oncentration gas-phase organic compounds. Anal. Chem..

[28] Ibeas I.L., Cuevas A.R., Andrikopoulou C., Person V., Baldas L., Colin S., Calvé S.L. (2019). Sub-ppb level detection of BTEX gaseous mixtures with a compact prototype GC equipped with a preconcentration unit. Micromachines.

[29] Garg A., Akbar M., Vejerano E., Narayanan S., Nazhandali L., Marr L.C., Agah M. (2015). Zebra GC: A mini gas chromatography system for trace-level determination of hazardous air pollutants. Sens. Actuators B Chem..

